# Targeting Ferroptosis as a Novel Approach to Alleviate Aortic Dissection

**DOI:** 10.7150/ijbs.72528

**Published:** 2022-06-21

**Authors:** Na Li, Xin Yi, Yi He, Bo Huo, Yue Chen, Zihao Zhang, Qunhui Wang, Yi Li, Xiaoxuan Zhong, Rui Li, Xue-Hai Zhu, Zemin Fang, Xiang Wei, Ding-Sheng Jiang

**Affiliations:** 1 Division of Cardiothoracic and Vascular Surgery, Sino-Swiss Heart-Lung Transplantation Institute, Tongji Hospital, Tongji Medical College, Huazhong University of Science and Technology, Wuhan, Hubei, China.; 2 Department of Cardiology, Renmin Hospital of Wuhan University, Wuhan, Hubei, China.; 3 Key Laboratory of Organ Transplantation, Ministry of Education; NHC Key Laboratory of Organ Transplantation; Key Laboratory of Organ Transplantation, Chinese Academy of Medical Sciences, Wuhan, Hubei, China.

**Keywords:** Aortic dissection, METTL3, Ferroptosis, SLC7A11, FSP1/AIFM2, Liproxstatin-1

## Abstract

A variety of programmed cell death types have been shown to participate in the loss of smooth muscle cells (SMCs) during the development of aortic dissection (AD), but it is still largely unclear whether ferroptosis is involved in the development of AD. In the present study, we found that the expression of key ferroptosis regulatory proteins, solute carrier family 7 member 11 (SLC7A11), ferroptosis suppressor protein 1 (FSP1) and glutathione peroxidase 4 (GPX4) were downregulated in aortas of Stanford type A AD (TAAD) patients, and liproxstatin-1, a specific inhibitor of ferroptosis, obviously abolished the β-aminopropionitrile (BAPN)-induced development and rupture of AD in mice. Furthermore, the expression of methyltransferase-like 3 (METTL3), a major methyltransferase of RNA m^6^A, was remarkably upregulated in the aortas of TAAD patients, and the protein levels of METTL3 were negatively correlated with SLC7A11 and FSP1 levels in human aortas. Overexpression of METTL3 in human aortic SMCs (HASMCs) inhibited, while METTL3 knockdown promoted SLC7A11 and FSP1 expression. More importantly, overexpression of METTL3 facilitated imidazole ketone erastin- and cystine deprivation-induced ferroptosis, while knockdown of METTL3 repressed ferroptosis of HASMCs. Overexpression of either SLC7A11 or FSP1 largely abrogated the effect of METTL3 on HASMC ferroptosis. Therefore, we have revealed that ferroptosis is a critical cause of AD in both humans and mice and that METTL3 promotes ferroptosis of HASMCs by inhibiting the expression of SLC7A11 and FSP1. Thus, targeting ferroptosis or m^6^A RNA methylation is a potential novel strategy for the treatment of AD.

## Introduction

Aortic dissection (AD) is an acute and highly fatal vascular condition with an annual incidence of 3.5-7.2 per 100 000 individuals 1, 2. In a recent 15-year follow-up study in Sweden, the out-of-hospital mortality was approximately 29%, and the 30-day in-hospital mortality was 21%-26% 1. AD is caused by tearing of the intimal and medial layers of the aorta resulting in the formation of true and false lumina 3. One of the main pathological features of AD is progressive medial degeneration of the aortic wall, which includes but is not limited to loss of smooth muscle cells (SMCs) and fragmentation of elastic fibers 4-6. Several types of programmed cell death have been reported to be involved in the death of SMCs during AD, such as apoptosis 7, 8.

Ferroptosis is a type of programmed cell death driven by the iron-dependent accumulation of lipid hydroperoxides 9. The most important antioxidant systems in cells include glutathione, nicotinamide adenine dinucleotide phosphate (NADPH), and coenzyme Q_10_ (CoQ_10_) 9. Depletion of antioxidant systems is an important cause of ferroptosis 10, 11. The system Xc^-^ (a complex composed of solute carrier family 7 member 11 (SLC7A11) and SLC3A2) pathway, glutathione-glutathione peroxidase 4 (GPX4) pathway, and NADPH-ferroptosis suppressor protein 1 (FSP1)-CoQ_10_ pathway are major pathways regulating ferroptosis 9. Moreover, there is growing evidence that autophagy is closely related to ferroptosis and that autophagy can promote ferroptosis by degrading ferritin 12. Our recent results demonstrated that the levels of a marker of autophagy, microtubule-associated protein 1 light chain 3 (LC3), were increased in the aortic tissues of AD patients, and autophagic cell death of aortic SMCs is critical for the development of AD 13, 14. However, whether ferroptosis contributes to SMC loss during AD is unknown.

N^6^-methyladenosine (m^6^A) RNA methylation has been reported to participate in various cardiovascular diseases 15. Methyltransferase-like 3 (METTL3) is the major methyltransferase that installs m^6^A on RNA molecules 16. Recently, METTL3 has been shown to be involved in the regulation of autophagy, but the role of METTL3 in autophagy is still controversial 17-19. Song *et al.* found that METTL3 inhibited autophagic flux in hypoxia/reoxygenation (H/R)-treated cardiomyocytes 18, while Liu *et al.* demonstrated that METTL3 positively regulated autophagy by promoting the expression of autophagy related 5 (ATG5) and ATG7 in non-small cell lung cancer (NSCLC) cells 17. These results indicate that the regulatory effect of METTL3 on autophagy may be treatment- or cell type-dependent. Although a published study has reported that m^6^A levels are higher in the aortic tissues of abdominal aortic aneurysm (AAA) patients than in healthy aortic tissues 20, the expression pattern and role of METTL3 in AD are unclear. METTL3 has a regulatory effect on autophagy 17, and autophagy is not only closely related to ferroptosis 12 but also involved in the development of AD 13. However, whether METTL3 affects AD through regulation of ferroptosis remains unknown.

In the present study, we found that SLC7A11, FSP1 and GPX4, key regulators of ferroptosis, were downregulated in the aortas of Stanford type A AD (TAAD) patients, and inhibition of ferroptosis by liproxstatin-1 largely abrogated β-aminopropionitrile (BAPN)-induced AD in mice. Furthermore, the expression level of METTL3 in aortic tissues of TAAD patients was significantly higher than that in non-AD subjects, and METTL3 promoted the degradation of the mRNAs of SLC7A11 and FSP1 to inhibit their protein expression in primary cultured human aorta SMCs (HASMCs). METTL3 accelerated imidazole ketone erastin (IKE)- and cystine deprivation-induced ferroptosis of HASMCs by suppressing SLC7A11 and FSP1 expression, and overexpression of SLC7A11 or FSP1 largely reversed the HASMC ferroptosis caused by METTL3 overexpression. These results indicate that reducing m^6^A methylation can suppress the ferroptosis of SMCs and inhibiting ferroptosis is a potential strategy to delay the pathological processes of AD.

## Methods and Materials

### Human aorta samples

A total of 146 ascending aortic tissue samples were collected from 81 patients diagnosed with TAAD and 65 subjects with non-AD. The non-AD samples were collected from patients who underwent heart transplantation at Tongji Hospital, Tongji Medical College, Huazhong University of Science and Technology. Patients were excluded who had a heritable form of aortopathy, such as Marfan syndrome, Loeys-Dietz syndrome, or iatrogenic TAAD. The clinical information is described in Supplemental Table S1. All patients signed an informed consent form. All experiments involving human specimens in this study were approved by the ethics committee of Tongji Hospital, Tongji Medical College, Huazhong University of Science and Technology. For western blot assay, 35 non-AD samples and 67 TAAD samples were used, and 30 non-AD and 60 TAAD ascending aortic tissues were included to make a tissue chip for immunohistochemical staining. Among them, 46 TAAD samples were used for both western blot and immunohistochemical staining.

### Mouse AD model

All animal experiments were approved by the Institutional Animal Care and Use Committee at Tongji Hospital, Tongji Medical College, Huazhong University of Science and Technology. The mouse AD model was established by using 0.6% (w/vol) BAPN (A3134, Sigma-Aldrich) to treat 3-week-old male mice for 3 weeks in our study 21. In detail, 0.6 g of BAPN was dissolved in 100 mL of distilled water, and the mice were allowed to drink the treated water freely. Liproxstatin-1 (5 mg/kg, S7699, Selleck) was injected intraperitoneally into BAPN-treated mice once every 2 days in the first week and once a day in the second and third weeks. The condition of the mice was monitored every day, and the dead mice were dissected to confirm whether they died of AD rupture. After 3 weeks of BAPN treatment, ultrasonography was performed on the surviving mice, and the maximum diameters of the ascending aorta, aortic arch, and descending aorta were measured. Finally, the mice were sacrificed, and aortic samples were collected from the mice for follow-up research.

### Plasmids

The full-length human METTL3, SLC7A11 and FSP1 CDSs were amplified by PCR and cloned into the pHAGE lentiviral vector. The primers used to amplify the CDSs of METTL3, SLC7A11 and FSP1 were as follows: METTL3 forward primer: 5'-CCGACGCGTGCCACCATGTCGGACACGTGGAG-3', METTL3 reverse primer: 5'-ACGCGTCGACTAAATTCTTAGGTTTAGAGATGATAC-3'; SLC7A11 forward primer: 5'-CCGACGCGTGCCACCATGGTCAGAAAGCCTGTTGTGTCC-3', SLC7A11 reverse primer: 5'-TCGGTCGACTAACTTATCTTCTTCTGGTACAACTTCCAG-3'; FSP1 forward primer: 5'-TCGGGTTTAAACGGATCCGCCACCATGGGGTCCCAGGTCTCGGTGGAAT-3', FSP1 reverse primer: 5'-GGGCCCTCTAGACTCGAGTCAAGGTGGAGACTGCCTCATGG-3'. Short hairpin RNA (shRNA) fragments targeted to human METTL3 were inserted into pLKO.1 vector, and the target sequences were as follows: shRNA#1, 5'-GCTGCACTTCAGACGAATTAT-3'; shRNA#2, 5'-GCCAAGGAACAATCCATTGTT-3'.

### Cell culture and treatments

Primary cultured human aortic smooth muscle cells (HASMCs) were derived from aortic tissue trimmed from heart donors during transplantation surgery 14. After removing the intima and adventitia of the aortic tissue in DME/F12 medium (SH30023.01; HyClone) on ice, the tissue was cut into pieces of approximately 1.5 mm^2^, and the pieces were transferred to a new petri dish. The tissue pieces were distributed evenly on the bottom of the petri dish and left to stand for 30 min. Then, an appropriate amount of DME/F12 medium supplemented with 10% fetal bovine serum (FBS; 1767839; Thermo Fisher Scientific) and 1% penicillin-streptomycin (15140-122; Thermo Fisher Scientific) was added. Long, spindle-shaped SMCs grew out from the tissue blocks after the blocks were cultured in an incubator for approximately 7 days. After that, the HASMCs were routinely passaged for further experiments when the confluence reached approximately 80%. Lentiviruses carrying the indicated vectors were used to infect the HASMCs for the following experiments. Ferroptosis of HASMCs was induced with 2 μM imidazole ketone erastin (IKE, S8877; Selleck) or by cystine deprivation. After infected with the lenti-Flag or lenti-METTL3 for 48h, HASMCs were treated with 5 μg/mL Actinomycin D (ACTD, S8964; Selleck) for indicated times to investigate mRNA degradation. The numbers of cells were counted by using a Cellometer Mini (Nexcelom Bioscience).

### Cell viability assay

Cell viability was evaluated by using Cell Counting Kit-8 (CCK-8) and lactate dehydrogenase (LDH) release assays as reported previously 13, 14. A total of 8000 HASMCs were seeded in each well of a 96-well plate. After treatment with IKE or cystine deprivation for the indicated times, the HASMCs were incubated with CCK-8 reagent (CK04; Dojindo), and then the absorbance was detected at 450 nm. Furthermore, a cytotoxicity LDH assay kit (CK12; Dojindo) was also used to evaluate HASMC injury under the indicated treatments. The optical density (OD) value was measured by using a microplate spectrophotometer at 490 nm wavelength.

### Western blot analysis

Western blotting was performed as previously reported 22-24. Briefly, total proteins were extracted from human aorta samples or HASMCs by using radioimmunoprecipitation assay (RIPA) lysis buffer. The proteins were separated by SDS-PAGE and then transferred to a polyvinylidene fluoride (PVDF) membrane. After that, the membrane was blocked with 5% nonfat milk for 90 min and then incubated with the indicated primary antibody overnight at 4 °C. The primary antibodies used in this study were as follows: ALKBH5 (HPA007196, Atlas Antibodies), AIFM2/FSP1 (HPA042309, Atlas Antibodies), METTL14 (HPA038002, Atlas Antibodies), METTL3 (15073-1-AP, Proteintech), WTAP (60188-1-Ig, Proteintech), SLC3A2 (15193-1-AP, Proteintech), FTO (ab92821, Abcam), GPX4 (ab125066, Abcam), β-Actin (#8457, Cell Signaling Technology), SLC7A11 (NB300-318, Novus Biologicals), and Flag (F1804, Sigma-Aldrich) antibodies.

### Histological analysis and immunohistochemical staining

Thirty non-AD and sixty TAAD aortic tissues were fixed in formaldehyde, dehydrated, and then embedded in paraffin to make a tissue chip. Hematoxylin and eosin (HE) staining, elastin Verhoeff-van Gieson (EVG) staining and immunohistochemical staining were performed on 5 μm sections as previously described 22. For immunohistochemical staining, after baking the slices, xylene dewaxing, and gradient alcohol hydration, antigen retrieval was achieved in ethylenediaminetetraacetic acid (EDTA) solution at high temperature for 20 min. Subsequently, the slices were incubated with the indicated primary antibody (against METTL3 (15073-1-AP, Proteintech, 1:500), SLC7A11 (NB300-318, Novus Biologicals, 1:200), FSP1 (HPA042309, Atlas Antibodies, 1:100), HMOX1 (10701-1-AP, Proteintech, 1:200), TFR (13-6800, Invitrogen, 1:200), or 4-HNE (MAB3249, R&D Systems, 1:2000) overnight at 4 °C. The primary antibody was washed off with phosphate-buffered saline (PBS), after which polymer enhancer (Kit-9902, MXB Biotechnologies) and the corresponding secondary antibody were added. A DAB horseradish peroxidase color development kit was used to develop color.

### Determination of iron level

The ferrous iron (Fe^2+^) content was detected by using an iron assay kit (MAK025, Sigma-Aldrich) according to the manufacturer's instructions. Briefly, HASMCs were rapidly homogenized in 4 volumes of iron assay buffer. After centrifugation at 16,000 × g for 10 min at 4 °C, 100 μL of supernatant was added to the sample wells in a 96-well plate. After incubating for 30 min at 25 °C in the dark, 100 μL of iron probe was added to each well containing standard or test sample. The reaction was incubated for 60 min at 25 °C in the dark after thorough mixing. The absorbance was measured at 593 nm. The similar procedure was performed to detect the serum ferrous iron levels of mice treated indicated stimulus.

### Malondialdehyde (MDA) assay

The MDA content in cells was detected by using an MDA assay kit (S0131, Beyotime) according to the instructions. Briefly, HASMCs that subjected to the indicated treatments were lysed with RIPA buffer. The samples were centrifuged at 12,000 × g for 15 min. Then, 100 µL of the supernatant was taken from each sample to react with MDA working solution, which was composed of thiobarbituric acid (TBA) diluent, TBA storage solution and antioxidants. After reacting at 100 °C for 15 min, the mixture was cooled to room temperature in a water bath and centrifuged at 1,000 × g for 10 min, and 100 µL of the supernatant was collected for absorbance detection at 532 nm.

### Lipid peroxidation assay

The BODIPY-C11 kit (D3861, Thermo Fisher) was used to evaluate lipid reactive oxygen species (ROS) levels following the procedure of the manual 25. After indicated treatments, HASMCs were washed with PBS to remove dead cells and then collected. The cell culture medium containing 5 µM BODIPY-C11 was added into cells and the mixture was incubated for 30 min at 37 °C. After washing three times with PBS, the excess sensor was removed and the fluorescence emission peak from 590 nm to 510 nm was measured by flow cytometry using a BD FACS Aria cytometer (BD Biosciences). The ratio of BODIPY-C11 oxidation (590 nm/510 nm) is proportional to the levels of lipid ROS.

### GEO dataset collection and analysis

In the present study, we retrieved the human TAAD datasets in the GEO database, and finally selected the GSE153434 dataset for bioinformatics analysis, which contains largest number of samples among all the transcriptome datasets. There are 10 normal ascending aortic tissue samples and 10 TAAD tissue samples in the GSE153434 (Platform: GPL20795) 26. Principal component analysis (PCA) was performed to reveal the significantly different composition of TAAD and normal aortic tissue samples based on the raw count data of GSE153434. Then, we screened the differentially expressed genes (DEGs) between the TAAD and normal samples by the DESeq2 package (version 1.26.0) in R software (version 3.6.1) 27. The |fold change| ≥1.5 and adjust p value ≤ 0.05 were defined as the thresholds for selecting the DEGs. In addition, RIdeogram package (version 0.2.2) was used to display the distribution of DEGs on the chromosomes 28. The clusterProfiler package (version 3.14.3) was used to perform the GO and Kyoto Encyclopedia of Genes and Genomes (KEGG) enrichment analyses for DEGs and *p*-value < 0.05 was set as the threshold for the enriched GO terms and significant pathways 29.

To explore the regulation effect of METTL3 on the m^6^A methylation levels of SLC7A11 and FSP1, we downloaded the METTL3-knockout (METTL3-KO) datasets (GSE110320 and GSE165863) from GEO database. In GSE110320 dataset, Huang *et al.* revealed the landscape of N6-methyladenosine in HepG2 cells with or without METTL3-knockout by MeRIP-seq based on GPL20301 platform 30. As for GSE165863 (Platform: GPL21273), five mouse hematopoietic cell types (GMP, MEP, MPP, ST-HSC, LT-HSC) were used to uncover the alterations of m^6^A modification with or without METTL3-knockout 31. Then, m^6^A methylation levels of SLC7A11 and FSP1 were compared between METTL3-KO and control groups.

### Statistical analysis

All the data are presented as the mean ± standard deviation (SD) in the present study. For human sample study, Student's two-tailed t-test was used to compare the means of two groups. Correlations between variables were analyzed by Pearson correlation analysis. Multiple group comparisons were performed by using one-way ANOVA with post hoc analysis. All statistical analyses were performed in SPSS software (version 13.0), and *p <* 0.05 was considered to indicate statistical significance.

## Results

### Ferroptosis was activated during TAAD development in human

Our previously published results demonstrated that autophagic cell death was critical for SMCs loss during AD development, and apoptosis was also found to be involved in this pathological process 13, 14. To further investigate whether there are other types of programmed cell death also participates in the development of AD, we first analyzed a RNA-sequence dataset of aorta with or without TAAD in human downloaded from GEO database. The results of PCA analysis showed that clearly separated the control group from TAAD group after normalized RNA-sequence expression values (Supplemental Figure S1A). According to the threshold of |fold change| ≥1.5 and adjust p value ≤ 0.05, a total of 1870 differential expression genes were included, of which 885 genes were upregulated and 985 genes were downregulated, which were shown on chromosomes (Supplemental Figure S1B). Enrichment analysis of GO and KEGG revealed that SMC contraction, extracellular matrix regulation, inflammatory signaling pathways, and cell death were highlighted (Supplemental Figure S1C). From KEGG analysis, we found that apoptosis, autophagy, necroptosis and ferroptosis were the potential programmed cell deaths involved in the development of AD, especially apoptosis and ferroptosis were highlighted (Supplemental Figure S1C). The differential expression genes related to these programmed cell deaths were shown in Supplemental Figure S1D.

**Figure 1:**
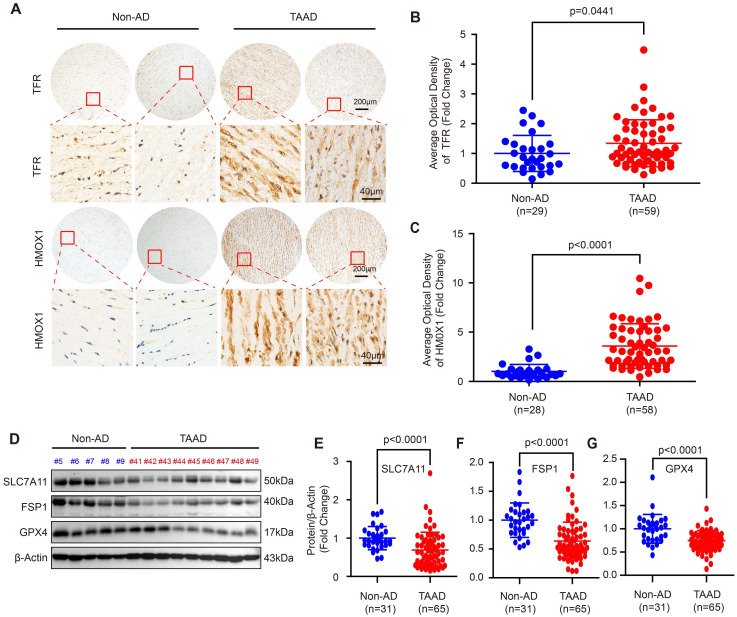
** Ferroptosis was involved in the development of AD in human. (A)** Immunohistochemical staining of TFR and HMOX1 in aortas of patients with non-AD and TAAD. **(B and C)** Quantitative of the average optical density of TFR (B) (n=29 non-AD and n=59 TAAD) and HMOX1 (C) (n=28 non-AD and n=58 TAAD) in (A). **(D)** Representative western blots of SLC7A11, FSP1 and GPX4 in the aortas of non-AD and TAAD patients (n=31 non-AD and n=65 TAAD). **(E-G)** Quantitative results of SLC7A11 (E), FSP1 (F), and GPX4 (G) expression in the aorta. β-Actin served as the loading control.

Since the role of apoptosis, autophagy and necroptosis in AD has been widely investigated 5, 7, 13, 26, we focused on ferroptosis in this study. To further confirm ferroptosis involvement in AD, we first detected the expression levels of the ferroptosis-related molecules, transferrin receptor (TFR) and heme oxygenase 1 (HMOX1). Compared with non-AD control, both TFR and HMOX1were remarkably increased in aortas of patients with TAAD (Figure 1A-1C). More importantly, the protein levels of SLC7A11, FSP1 and GPX4, which were key molecules in the three main pathways regulating ferroptosis, were detected in aortic tissues of non-AD and TAAD patients. Our results showed that compared with those of non-AD patients, the aortas of TAAD patients exhibited downregulated protein levels of SLC7A11, FSP1 and GPX4 (Figure 1D-1G and Supplemental Figure S2). These results indicated that aberrant expression of HMOX1, TFR, SLC7A11, FSP1 and GPX4 may active ferroptosis to contribute to the development of AD.

### Reductions of BAPN-induced AAD development and aortic degeneration in mice treated with liproxstatin-1, an inhibitor of ferroptosis

**Figure 2:**
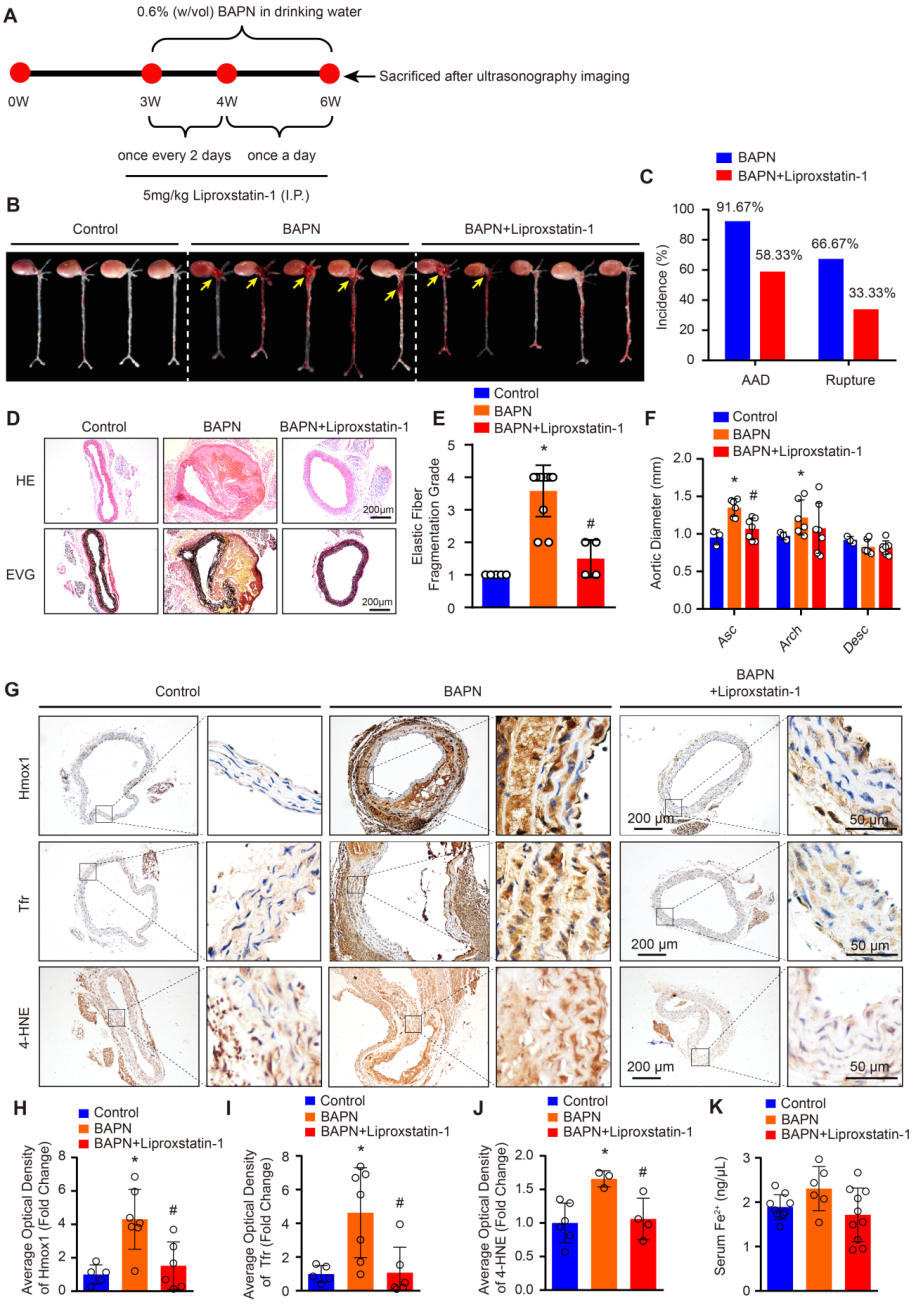
** Liproxstatin-1, an inhibitor of ferroptosis, largely abolished BAPN-induced AD development. (A)** Flow chart of the animal experiments.** (B)** Representative images of excised aortas of the indicated groups (n=12 per group). **(C)** The overall incidences of AD were significantly lower in mice challenged with BAPN+liproxstatin-1 (AAD occurred in 7 (4 ruptured) out of 12 mice) than in mice challenged with BAPN alone (AAD occurred in 11 (8 ruptured) out of 12 mice) (n=12 per group). **(D)** Representative hematoxylin and eosin (HE) staining and elastin Verhoeff-van Gieson (EVG) staining of aortas (n=5 control; n=12 BAPN; n=4 BAPN+Liproxstatin-1). **(E)** Quantification of aortic elastic fiber fragmentation based on EVG staining in the indicated groups (n=5 control; n=12 BAPN; n=4 BAPN+Liproxstatin-1).** (F)** The mean aortic diameters of various aortic segments were measured based on ultrasonography images; Asc indicates ascending aorta; Arch indicates aortic arch; Desc indicates descending aorta (n=3 control; n=6 BAPN; n=7 BAPN+Liproxstatin-1). **(G-J)** Representative immunohistochemical staining of Hmox1, Tfr and 4-HNE (G); and their average optical density in aortic tissues of mice treated with or without BAPN or liproxstatin-1 (H-J) (n=5-6 control; n=3-7 BAPN; n=4-6 BAPN+Liproxstatin-1). **(K)** The serum ferrous iron levels in the indicated groups (n=9 control; n=6 BAPN; n=10 BAPN+Liproxstatin-1). *p<0.05 vs control; #*p<*0.05 vs BAPN.

The protein levels of SLC7A11, FSP1 and GPX4 are strikingly reduced in patients with TAAD, and reductions in their levels have been shown to promote ferroptosis 15. Thus, we hypothesized that ferroptosis of SMCs may be crucial in the pathological process of AD. To validate this hypothesis, we treated BAPN-induced mice with the ferroptosis-specific inhibitor liproxstatin-1 to investigate the effect of ferroptosis on AD (Figure 2A). In 3-week-old mice, the incidence of BAPN-induced aortic aneurysm and dissection (AAD) was 91.67%, and the mortality due to dissection rupture was 66.67% after BAPN treated for 3 weeks (Figure 2B and 2C). All occurrences of AAD were in the ascending aorta, aortic arch, and thoracic aorta (Figure 2B). The results of HE and EVG staining showed that BAPN treatment for 3 weeks induced obvious medial degeneration of the aorta, SMC loss, and breakage of elastic fibers in the aorta in young mice (Figure 2D and 2E). In addition, compared with mice given normal drinking water, mice given BAPN-treated drinking water exhibited larger ascending aorta and aortic arch diameters (Figure 2F). More importantly, liproxstatin-1, an inhibitor of ferroptosis, strikingly reduced the incidence and mortality of BAPN-induced AAD in mice (Figure 2B and 2C). Liproxstatin-1 also protected the mice from BAPN-induced medial degeneration and fragmentation of elastin in the aorta and from aortic dilatation (Figure 2D-2F). In addition, we further evaluated the expression levels of ferroptosis-related molecules, Hmox1 and Tfr, and lipid peroxidation (4-hydroxynonenal, 4-HNE) in mice via immunohistochemical staining. In untreated mice, a low levels of Hmox1, Tfr and 4-HNE were detected in the aorta, but after treatment with BAPN, the levels of Hmox1, Tfr and 4-HNE were significantly increased (Figure 2G-2J). Liproxstatin-1 largely attenuated the increase in Hmox1, Tfr and 4-HNE induced by BAPN (Figure 2G-2J). In addition, the serum content of ferrous ions was slightly elevated in mice treated with BAPN, which was reversed by liproxstatin-1 treatment (Figure 2K).

These results indicate that ferroptosis is one of the main mechanisms resulting in medial degeneration and also a programmed cell death leading to aortic SMCs loss during the process of AD. Thus, inhibiting the ferroptosis of SMCs is a potentially effective therapeutic strategy to reduce the occurrence of AD.

### Expression levels of m^6^A modulators in aortic tissues with or without TAAD

Recently, Ma* et al.* found that m^6^A-modified SLC7A11 mRNA was bound by the m^6^A reader YT521-B homology domain-containing 2 (YTHDC2) to promote SLC7A11 mRNA decay and suppress its expression during lung adenocarcinoma 32. In recent years, several studies have confirmed that RNA m^6^A methylation is related to aortic diseases, especially AAA 20, 33. For example, He *et al.* found that m^6^A levels were significantly higher in AAA tissue samples than in normal aortic tissue samples, and a high level of m^6^A suggests an elevated risk of aneurysm rupture 20. However, whether RNA m^6^A methylation is involved in the regulation of SLC7A11, FSP1 and GPX4 expression and the pathological processes of AD is unclear. Although AAA and AD have many similarities in pathological mechanisms, there are still very large differences between these two vascular conditions. Thus, it is necessary to further investigate the biological function of RNA m^6^A methylation in AD.

To analyze the function of RNA m^6^A methylation in SLC7A11, FSP1 and GPX4 expression regulation and AD, we first detected the expression of m^6^A modulators, including m^6^A demethylases (AlkB homolog 5 (ALKBH5) and fat mass and obesity-associated protein (FTO)) and subunits of the methyltransferase complex (Wilms' tumor 1-associated protein (WTAP), METTL14, and METTL3), in a small cohort consisting of 12 non-AD and 28 TAAD aortic tissues. The results showed that compared with non-AD aortas, TAAD aortas exhibited downregulated protein levels of FTO but upregulated protein levels of METTL14 and METTL3 (Supplemental Figure S3A, S3C, S3E, and S3F). However, comparable expression levels of ALKBH5 and WTAP were detected between non-AD and TAAD samples (Supplemental Figure S3A, S3B, and S3D). These results indicate that RNA m^6^A methylation plays a potential role in the development of AD and that FTO, METTL14 and METTL3 may mediate this role.

### METTL3 is upregulated in aortas of patients with TAAD

**Figure 3:**
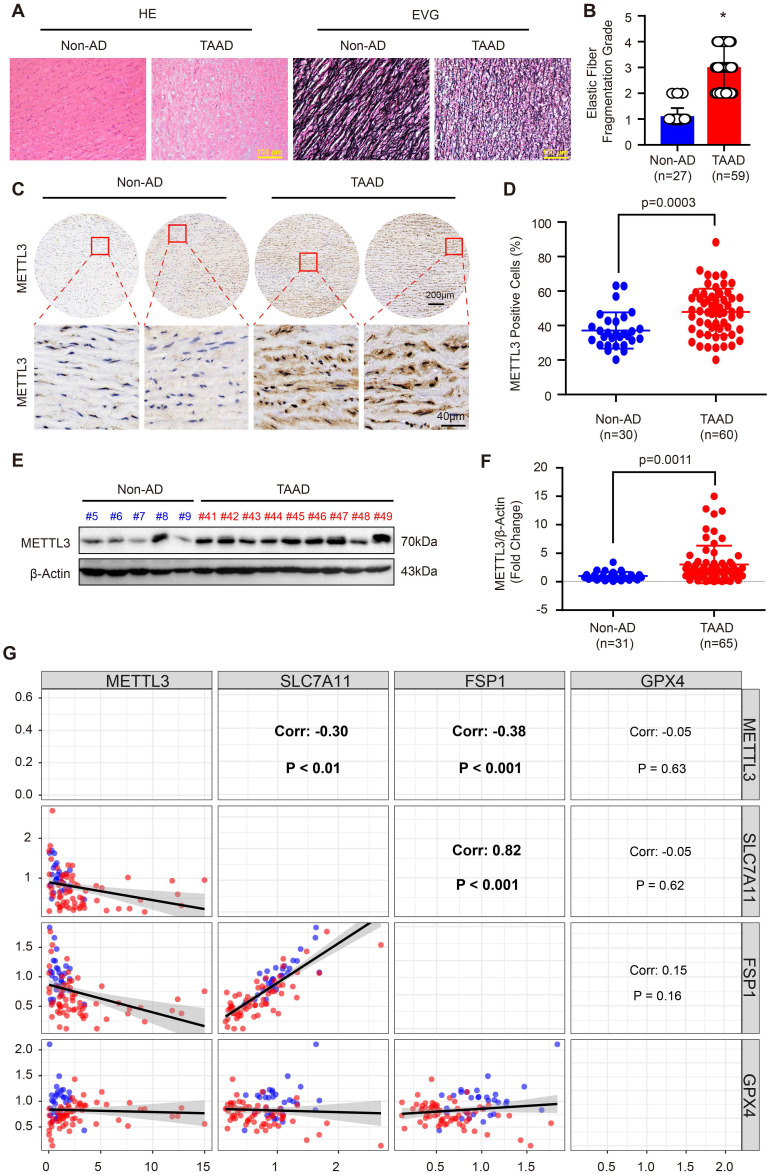
** METTL3 was remarkably upregulated in the aortas of TAAD patients. (A)** Representative hematoxylin and eosin (HE) staining and elastin Verhoeff-van Gieson (EVG) staining of aortic tissues of non-AD (n=27) and TAAD (n=59) patients. *p<0.05 vs non-AD. **(B)** Quantification of aortic elastic fiber fragmentation in non-AD and TAAD patients according to the EVG staining in (A). **(C and D)**. Representative immunohistochemical staining of METTL3 in an aortic tissue microarray including tissues from 30 non-AD and 60 TAAD patients (C). Percentages of METTL3 positive cells in the aortas of non-AD (n=30) and TAAD (n=60) patients (D). **(E and F)** The protein level of METTL3 in the aorta was evaluated by using western blot in 31 non-AD and 65 TAAD patients; **(E)** Representative western blots of METTL3; **(F)** Quantitative results for the METTL3 protein levels in (E). β-Actin served as the loading control. **(G).** Correlation of SLC7A11, FSP1, GPX4 and METTL3 protein expression in the aortas of human with or without TAAD (n=31 non-AD and n=65 TAAD patients).

Recently, Ma *et al.* reported that FTO contributes to AD via demethylation of Krüppel-like factor 5 (Klf5) 34. METTL3 and METTL14 are the subunits of the m^6^A methyltransferase complex, and METTL3 is the major subunit that exerts methyltransferase activity 15. Moreover, the results in Supplemental Figure S3 demonstrate that METTL14 and METTL3 have similar expression patterns and both of them were upregulated during AD. Thus, we focused on METTL3 in this study. To further validate the expression pattern of METTL3 in the aortas of non-AD and TAAD patients, a tissue microarray including aorta samples from 30 non-AD and 60 TAAD patients was performed. HE and EVG staining showed that compared with non-AD individuals, TAAD patients exhibited obvious medial degeneration and elastin fiber fragmentation (Figure 3A and 3B). As shown in Figure 3C, METTL3 was found to have increased expression in the aortas of TAAD patients, and METTL3 was mainly located in the nucleus and partly expressed in the cytoplasm (Figure 3C and 3D). Furthermore, we detected the protein levels of METTL3 in 31 non-AD and 65 TAAD aortic samples by using western blot analysis. The results showed that compared with those of non-AD patients, the aortas of TAAD patients had higher METTL3 protein levels (Figure 3E, 3F and Supplemental Figure S4), indicating a vital role of METTL3 in the pathological processes of TAAD.

### METTL3 negatively regulates SLC7A11 and FSP1 expression

**Figure 4:**
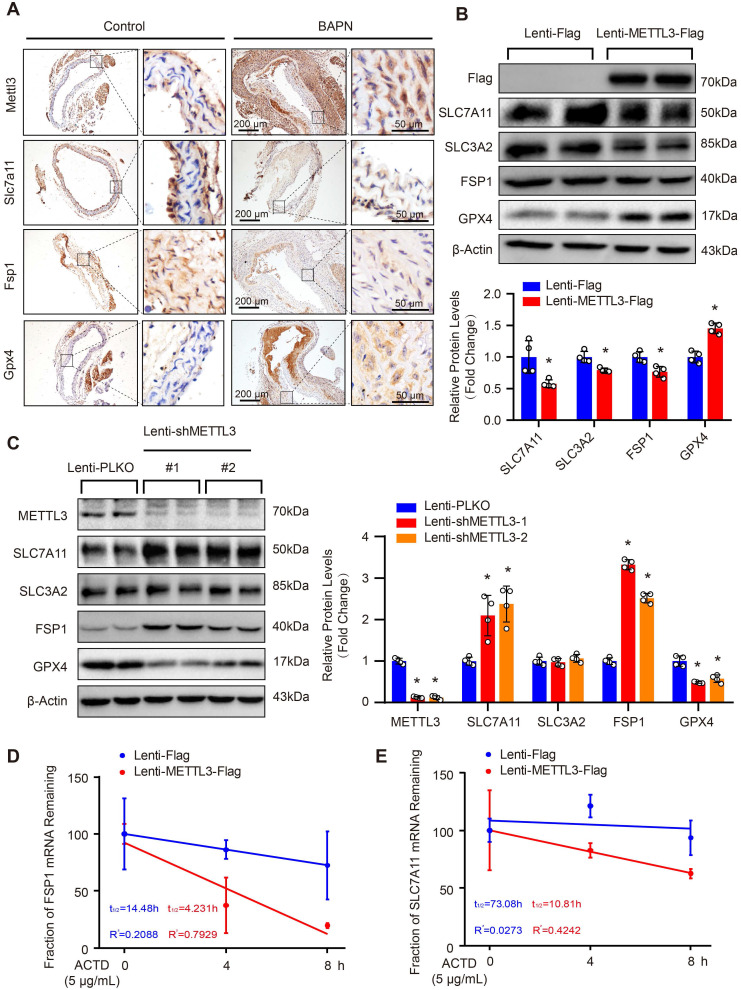
** METTL3 suppressed SLC7A11 and FSP1 expression via promoting their mRNA degradation. (A)** Representative immunohistochemical staining of Mettl3, Slc7a11, Fsp1 and Gpx4 in aortic tissues of mice treated with or without BAPN (n=6 control, n=3 BAPN). **(B and C)** The protein levels of METTL3 (Flag), SLC7A11, SLC3A2, FSP1, and GPX4 were detected by using western blot analysis in HASMCs infected with Lenti-Flag or Lenti-METTL3-Flag (B) or infected with Lenti-pLKO or Lenti-shMETTL3 (C) (n=4 per group). #1 and #2 indicate two different target sequences for knockdown of METTL3. The quantitative results are displayed. β-Actin served as the loading control. *p<0.05 vs Lenti-Flag or Lenti-pLKO control. **(D and E)** The fraction of FSP1 (D) or SLC7A11 (E) mRNA remaining in HASMCs with METTL3 overexpression or not after treated with 5 µg/mL Actinomycin D (ACTD) for indicated times (n=4 per group).

Since SLC7A11, FSP1 and GPX4 were decreased, while METTL3 was elevated in the aortic walls of patients with TAAD, we were very curious about whether METTL3, SLC7A11, FSP1 and GPX4 can form a pathway to participate in the regulation of the pathological process of AD. Intriguingly, although there is no significant correlation between GPX4 and METTL3, the expression of both FSP1 and SLC7A11 was significantly negatively correlated with the expression of METTL3 in the aorta, while SLC7A11 expression and FSP1 expression were strongly positively correlated (r=0.82, p<0.001) (Figure 3G). Similarly, the Mettl3 expression level was also enhanced by BAPN induction in mice (Figure 4A). In sharp contrast with the levels of Mettl3, the expression levels of Slc7a11 and Fsp1 were downregulated in the aortas of mice stimulated with BAPN (Figure 4A). Unexpectedly, Gpx4 showed opposite expression patterns in human and mouse during AD, Gpx4 protein level was elevated in aorta of mice treated with BAPN (Figure 4A). More importantly, overexpression of METTL3 significantly inhibited the expression of SLC7A11, SLC3A2 and FSP1, but promoted GPX4 expression in primary cultured HASMCs (Figure 4B). In contrast, in HASMCs with METTL3 knockdown, the expression of SLC7A11 and FSP1 was increased, while the protein level of GPX4 was decreased (Figure 4C). There was no significant change in the expression of SLC3A2 (Figure 4C). Since GPX4 has opposite expression patterns in human and mouse AD, and there is no significant correlation between GPX4 and METTL3 expression in human aortic samples, we focused on the regulatory relationship between METTL3 and SLC7A11/FSP1.

As METTL3 is an m^6^A methyltransferase, we are very curious whether METTL3 regulates mRNA m^6^A methylation of SLC7A11 and FSP1 to affect their protein expression. Thus, we first analyzed the MeRIP-seq datasets of human and mouse cell lines with METTL3-KO downloaded from the GEO database. In both human (GSE110320) and mouse (GSE165863) datasets, compared with control or wild-type (WT) cells, the m^6^A levels of SLC7A11 and FSP1 mRNA were decreased in METTL3-KO cells (Supplemental Figure S5). Since studies have reported that m^6^A methylation of SLC7A11 mRNA can promote its degradation 15, we used Actinomycin D (ACTD) treatment to inhibit mRNA synthesis and detect the effect of overexpression of METTL3 on the stability of SLC7A11 and FSP1 mRNA. Our results demonstrated that METTL3 overexpression accelerated the mRNA decay of both SLC7A11 and FSP1 in HASMCs (Figure 4D and 4E).

### METTL3 facilitates ferroptosis of HASMCs

**Figure 5:**
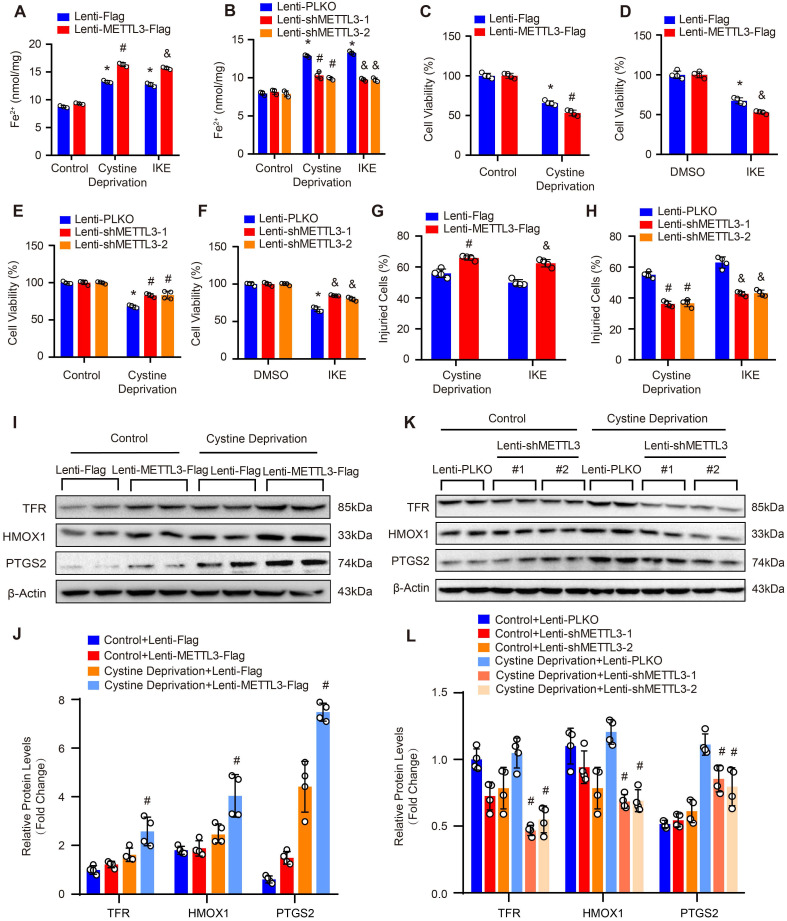
** METTL3 facilitated cystine deprivation- and IKE-induced ferroptosis of HASMCs. (A and B)** Content of ferrous ions (Fe^2+^) in HASMCs with METTL3 overexpression (A) or knockdown (B) treated with cystine deprivation or IKE (n=3 per group). **(C-F)** Cell viability was evaluated by using CCK-8 kit in HASMCs infected with Lenti-Flag, Lenti-METTL3-Flag, Lenti-pLKO or Lenti-shMETTL3 and treated with or without cystine deprivation or IKE (n=4 per group). **(G and H)** Cellular injury was measured by using an LDH kit in HASMCs infected with Lenti-Flag, Lenti-METTL3-Flag, Lenti-pLKO or Lenti-shMETTL3 under cystine deprivation or IKE treatment (n=4 per group).** (I-L)** The representative western blots of TFR, HMOX1, and PTGS2, and their quantitative results in HASMCs with METTL3 overexpression (I and J) or knockdown (K and L) (n=4 per group). β-Actin served as the loading control. *p<0.05 vs Lenti-Flag or Lenti-pLKO control; # p<0.05 vs Lenti-Flag or Lenti-pLKO with cystine deprivation; &p<0.05 vs Lenti-Flag or Lenti-pLKO with IKE treatment.

As SLC7A11 and FSP1 were the key factors regulating ferroptosis which is a programmed cell death characterized by iron-dependent lipid peroxidation 9, and METTL3 suppressed SLC7A11 and FSP1 expression, these results indicated that METTL3 may involve in the ferroptosis of SMCs. Thus, we further subjected primary cultured HASMCs to cystine deprivation or treated them with IKE, which are the most popular methods to induce ferroptosis 9. Compared with control, cystine deprivation or IKE treatment significantly increased the content of ferrous ions in HASMCs, and the concentration of ferrous ions was further elevated in HASMCs overexpressing METTL3 (Figure 5A) but decreased in HASMCs with METTL3 knockdown under the stimulation of cystine deprivation or IKE (Figure 5B). The results of CCK-8 and LDH release assays showed that METTL3 overexpression accelerated HASMC death induced by both cystine deprivation and IKE (Figure 5C, 5D and 5G). However, knockdown of METTL3 protected HASMCs from cystine deprivation- and IKE-induced ferroptosis (Figure 5E, 5F and 5H). Moreover, the expression of ferroptosis-related proteins TFR, HMOX1 and PTGS2 were induced by cystine deprivation treatment in HASMCs, which was further enhanced by METTL3 overexpression (Figure 5I and 5J). In contrast, METTL3 knockdown abrogated the expression levels of TFR, HMOX1 and PTGS2 under the treatment of cystine deprivation (Figure 5K and 5L).

**Figure 6:**
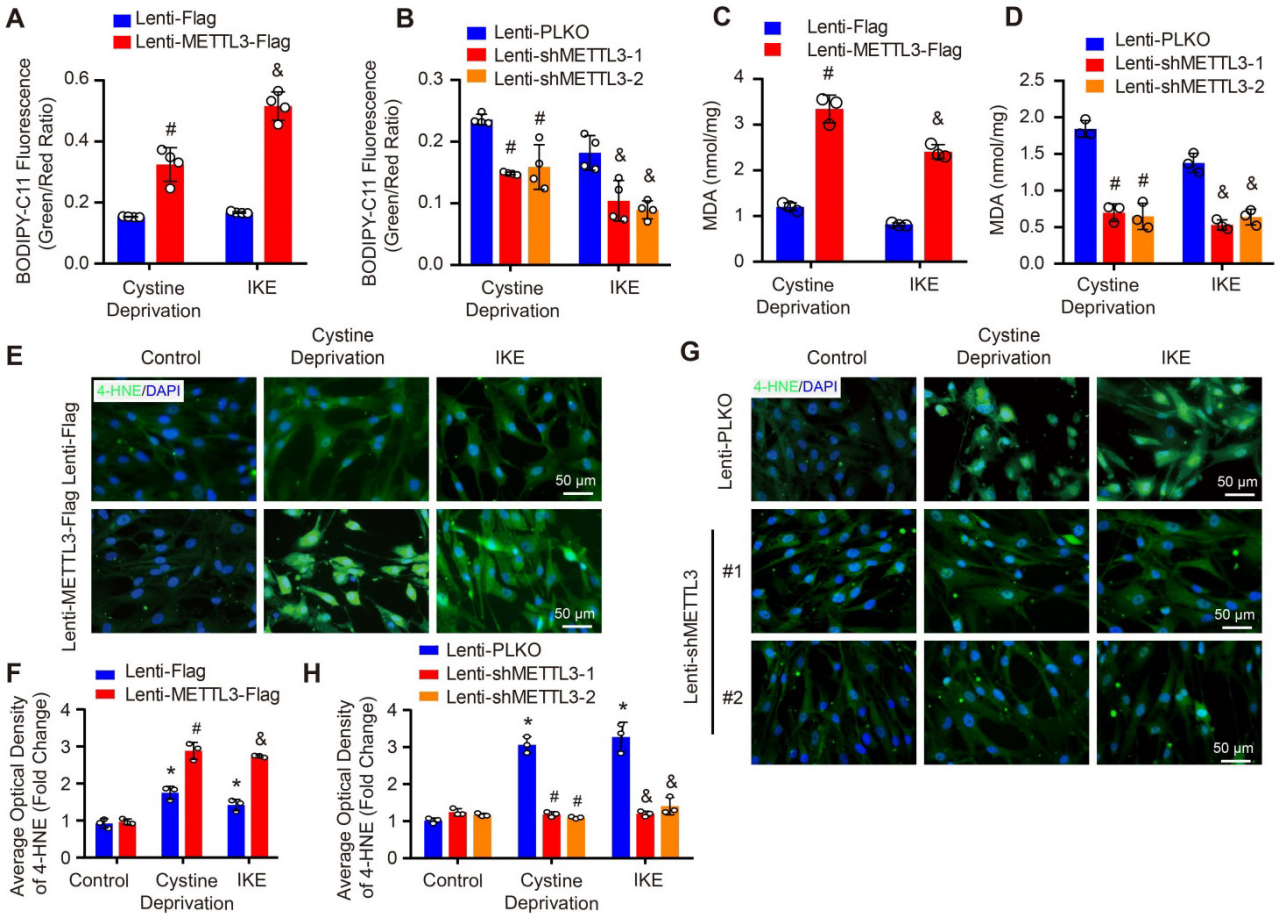
** METTL3 promoted cystine deprivation- and IKE-induced lipid peroxidation in HASMCs. (A and B)** The level of lipid ROS (oxidized BODIPY-C11 (green)/non-oxidized BODIPY-C11 (red) ratio) detected by using BODIPY-C11 kit in HASMCs with METTL3 overexpression (A) or knockdown (B) which treated with cystine deprivation or IKE (n=4 per group). **(C and D)** Lipid peroxidation was evaluated by using an MDA assay kit in HASMCs with the indicated treatments (n=3 per group).** (E-H)** Immunofluorescence staining of 4-HNE in HASMCs with METTL3 overexpression (E) or knockdown (G) which treated with cystine deprivation or IKE, and quantitative results are displayed in (F) and (H) (n=3 per group). *p<0.05 vs Lenti-Flag or Lenti-pLKO control; #* p<*0.05 vs Lenti-Flag or Lenti-pLKO with cystine deprivation; &*p<*0.05 vs Lenti-Flag or Lenti-pLKO with IKE treatment.

As we known, lipid peroxidation is a hallmark of ferroptosis. Thus, we further evaluated the impact of METTL3 on lipid peroxidation by using BODIPY-C11 fluorescent probe, MDA kit and 4-HNE staining. Our results showed that under both cystine deprivation and IKE treatments, the ratio of oxidized to non-oxidized lipids was obviously elevated in METTL3 overexpressed HASMCs, but reduced in HASMCs with METTL3 deficiency (Figure 6A and 6B). Moreover, the levels of MDA (the end product of lipid oxidation) and 4-HNE were also significantly increased in HASMCs overexpressing METTL3 (Figure 6C, 6E and 6F) but reduced in HASMCs with METTL3 knockdown (Figure 6D, 6G and 6H). These results further suggested that METTL3 can promote ferroptosis of HASMCs.

### Both SLC7A11 and FSP1 mediate the effect of METTL3 on ferroptosis in HASMCs

**Figure 7:**
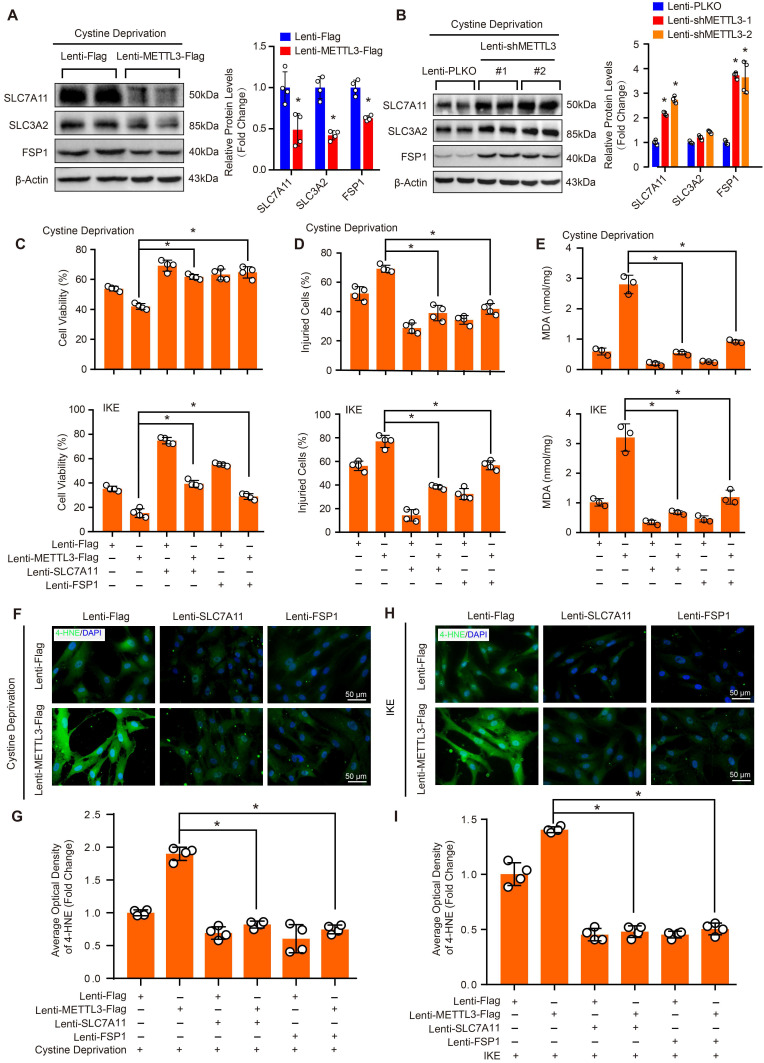
** SLC7A11 and FSP1 overexpression largely abrogated the effects of METTL3 on ferroptosis in HASMCs. (A and B)** The protein levels of SLC7A11, SLC3A2, FSP1, and GPX4 were detected by using western blot analysis in HASMCs infected with Lenti-Flag or Lenti-METTL3-Flag (A) or with Lenti-pLKO or Lenti-shMETTL3 (B) (n=4 per group). β-Actin served as the loading control.** (C)** Cell viability was evaluated by using CCK-8 kit in HASMCs with the indicated treatments (n=4 per group). **(D)** Cellular injury was measured by using an LDH kit in HASMCs with the indicated treatments (n=4 per group).** (E)** Lipid peroxidation was evaluated by using an MDA assay kit in HASMCs with the indicated treatments (n=3 per group). **(F-I)** Immunofluorescence staining of 4-HNE in HASMCs with the indicated treatments (F and H), and quantitative results are displayed in (G) and (I) (n=4 per group). *p<0.05 vs Lenti-Flag or Lenti-pLKO (A and B), or vs Lenti-METTL3 (C-I).

To further investigate the molecular mechanisms that mediate the function of METTL3 in ferroptosis in HASMCs, we evaluated the protein levels of SLC7A11, SLC3A2 and FSP1 in HASMCs with METTL3 overexpression or METTL3 knockdown under cystine deprivation treatment. The results showed that SLC7A11 and FSP1 expression was inhibited by METTL3 overexpression but enhanced after METTL3 knockdown, while comparable SLC3A2 protein levels were observed in HASMCs with or without METTL3 knockdown (Figure 7A and 7B). These results suggest that SLC7A11 and FSP1 likely mediate the regulatory effect of METTL3 on ferroptosis in HASMCs. To test this hypothesis, SLC7A11 or FSP1 was overexpressed in HASMCs with METTL3 overexpression to reverse cystine deprivation- and IKE-induced ferroptosis (Supplemental Figure S6). The results showed that either SLC7A11 or FSP1 overexpression significantly inhibited the ferroptosis of HASMCs induced by cystine deprivation or IKE, as evidenced by increased cell viability (Figure 7C), but reduced cell injury (Figure 7D), and lipid oxidation (Figure 7E-7I). More importantly, either SLC7A11 or FSP1 overexpression largely offset the impacts of METTL3 overexpression on ferroptosis of HASMCs treated with cystine deprivation or IKE, which was determined by cell viability, cell death, MDA content and 4-HNE levels (Figure 7C-7I). Collectively, these results demonstrate that METTL3 accelerates ferroptosis of HASMCs by suppressing SLC7A11 and FSP1 expression.

## Discussion

**Figure 8:**
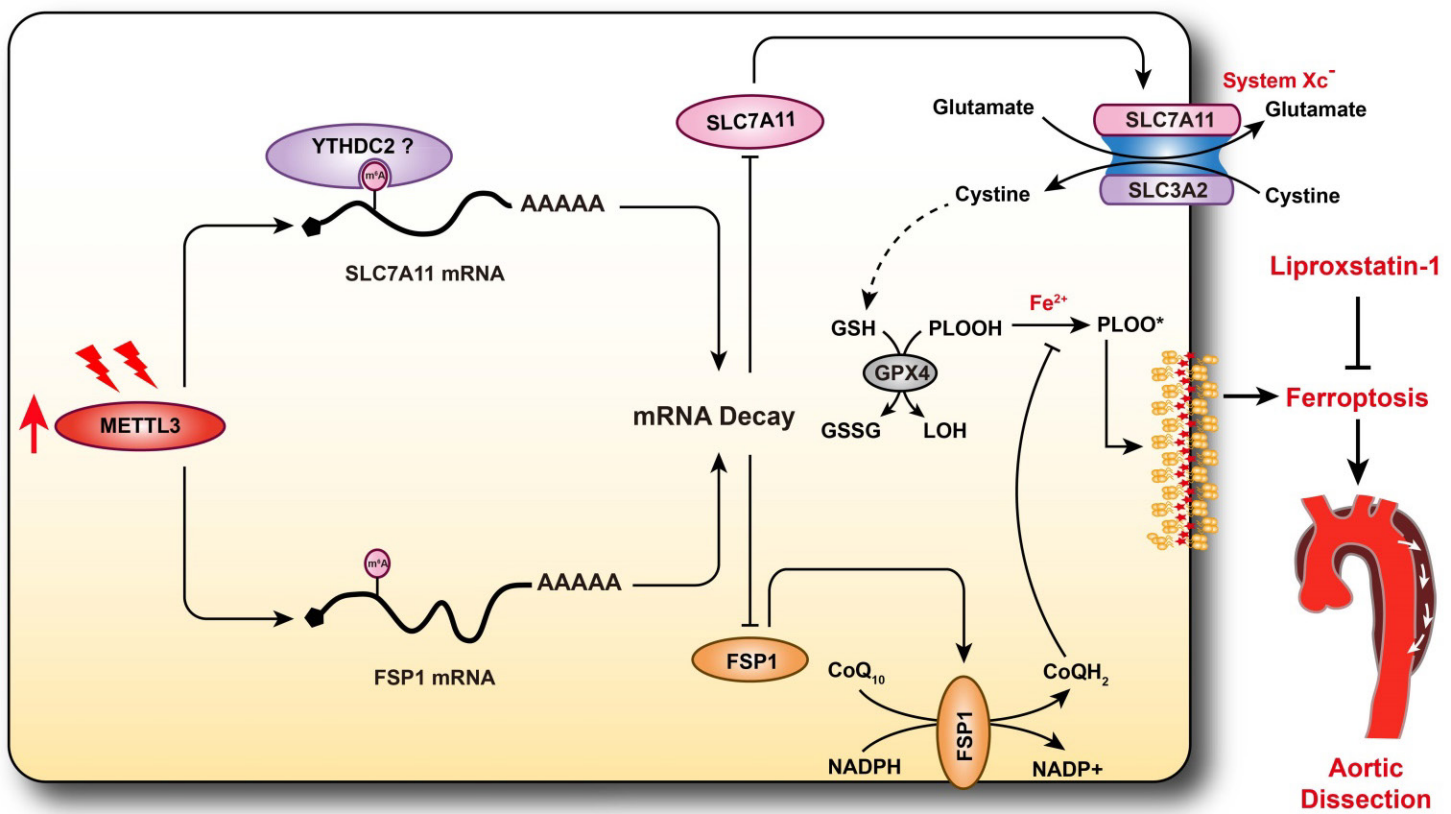
** Schematic summary.** Our results demonstrate that METTL3 is upregulated in the aortas of TAAD patients, and METTL3 overexpression facilitates ferroptosis of HASMCs by promoting the mRNA degradation of SLC7A11 and FSP1, then reducing their protein levels. Furthermore, ferroptosis is activated during the development of AD, and inhibition of ferroptosis by liproxstatin-1 largely abrogates BAPN-induced AAD in mice. These results suggest that inhibition of METTL3 or ferroptosis is an effective intervention strategy for AD.

AD is a serious vascular condition characterized by medial degeneration of the aorta 3. There is currently no effective drug for AD intervention or prevention. Recently, a growing body of evidence has suggested that programmed cell death plays central roles in SMC loss and AD 5, 7, 13, but the role of ferroptosis in AD remains unclear. In the present study, we revealed that ferroptosis was activated during the development of AD, and pharmacologically inhibiting ferroptosis with liproxstatin-1 partially alleviated aortic degeneration and AD development in mice. SLC7A11 and FSP1, the key regulators of ferroptosis, were downregulated, while the RNA methyltransferase METTL3 was obviously upregulated in aortas of TAAD patients. Furthermore, METTL3 negatively correlated with the expression of SLC7A11 and FSP1 in the aortas of human. Overexpression of METTL3 accelerated cystine deprivation- and IKE-induced ferroptosis by regulating SLC7A11 and FSP1 expression. These findings suggest that ferroptosis is a novel, critical programmed cell death involved in aortic degeneration and AD formation. We also found that the METTL3-SLC7A11/FSP1 axis regulates SMC ferroptosis (Figure 8).

RNA m^6^A methylation has been found to participate in several cardiovascular conditions, such as cardiac hypertrophy and I/R injury 18, 35. Recently, Ma* et al.* reported that the RNA m^6^A demethylase FTO promotes the proliferation and migration of SMCs 34. However, the vital roles of modulators of RNA m^6^A methylation in AD have remained unclear. Therefore, we first evaluated the expression levels of writers and erasers of m^6^A in non-AD and TAAD human aortic samples. We found that compared with non-AD samples, TAAD samples exhibited significant upregulation of METTL3 but downregulation of FTO, indicating that the level of RNA m^6^A methylation may increase during the pathological process of AD. Interestingly, the results of multiple independent research teams have demonstrated that m^6^A methylation levels are markedly increased in several cardiovascular diseases, including heart failure, cardiac hypertrophy, myocardial infarction, and abdominal aortic aneurysm 18, 20, 36, 37. These results suggest that increased RNA m^6^A methylation may be a hallmark of cardiovascular disease. Furthermore, METTL3 has been shown to promote Ang II- and CaCl_2_-induced abdominal aortic aneurysm by accelerating primary microRNA-34a maturation and inhibiting SIRT1 expression 33, and METTL3 also exacerbates oscillatory stress-induced atherogenesis 38. Inhibition of METTL3 or targeting of m^6^A methylation might be a potential strategy for intervention in cardiovascular diseases. Therefore, elucidating the function and downstream molecular mechanism of METTL3 or m^6^A methylation in AD will be conducive to precise intervention in AD.

Our previously published results demonstrate that autophagy is enhanced in the aortas of TAAD patients and that the histone methyltransferases EZH2 and EHMT2 participate in AD by regulating the autophagic cell death of SMCs 13, 14. In NSCLC cells, METTL3 enhances autophagy by upregulating the expression of ATG5 and ATG7 17. However, *Song et al.* found that METTL3 reduces H/R-induced autophagic flux in cardiomyocytes by regulating transcription factor EB (TFEB) 18. In recent years, autophagy has been reported to be closely related to ferroptosis, which is a newly identified iron-dependent programmed cell death pathway 9, 39. Since the expression of METTL3 is upregulated in the aortas of AD patients, and iron levels have also been reported to be increased in the aortas of AD patients 40, we were very curious about whether METTL3 affects ferroptosis of SMCs. We found that METTL3 accelerates cystine deprivation- and IKE-induced SMC ferroptosis and that this effect is abrogated by overexpression of SLC7A11 or FSP1. SLC7A11 is a component of the cystine/glutamate antiporter (xCT), which imports cystine for glutathione biosynthesis and antioxidant defense 41. SLC7A11 deficiency promotes ferroptosis by reducing glutathione synthesis 42. The m^6^A modification of mRNA is known to affect gene expression in various ways, such as decay, stability, translation, splicing, and nuclear export 15, 43. Our results revealed that METTL3 accelerates the mRNA degradation of SLC7A11 and suppresses its expression in HASMCs, which may be dependent on m^6^A in SLC7A11 mRNA. Ma* et al.* found that the m^6^A reader YTHDC2 promotes SLC7A11 mRNA decay by binding to m^6^A-modified SLC7A11 mRNA 32. Consistent with our results, Lin *et al.* also showed that knockout of METTL3 in pre-osteoblastic MC3T3-E1 cells attenuated the level of ferroptosis 44. However, Xu *et al.* revealed that METTL3-mediated m^6^A modification of SLC7A11 mRNA was read by YTHDF1, which promoted the translation of SLC7A11 mRNA to inhibit lung adenocarcinoma cell ferroptosis 45. The possible reason for this opposite scenario is that there are differences in the energy metabolism of tumor cells (aerobic glycolysis) and normal cells (mitochondrial oxidative phosphorylation) 46. On the other hand, the m^6^A methylated mRNAs may have different fates when recognized by different readers. For example, the mRNA of SLC7A11 is recognized by YTHDC2 to promote its degradation 32, while the recognition by YTHDF1 promotes its translation 45.

FSP1 (also known as apoptosis-inducing factor mitochondria-associated 2 (AIFM2)) was originally found to be a flavoprotein that regulates apoptosis, and it has recently been found to reduce lipid peroxidation by catalyzing the regeneration of CoQ_10_, which uses NAD(P)H to inhibit ferroptosis 47, 48. Our results showed that FSP1 was negatively regulated by METTL3 in HASMCs, and METTL3 overexpression promoted FSP1 mRNA degradation, but whether the mRNA of FSP1 is m^6^A-methylated and recognized by YTHDC2 requires more in-depth research.

Although we have demonstrated that METTL3 regulates ferroptosis and that ferroptosis is involved in the development of AD, there is still a lack of* in vivo* evidence to clarify the role of METTL3 in AD. Recently, Yankova* et al.* identified a highly potent and selective first-in-class catalytic inhibitor of METTL3, STM2457, which reduces acute myeloid leukemia growth and increases differentiation and apoptosis by selectively reducing m^6^A levels on known leukemogenic mRNAs 49. Given that RNA m^6^A levels are significantly elevated in patients with cardiovascular diseases such as AD, inhibitors of METTL3 may have a wide range of applications in this field.

The death of SMCs is one of the main causes of medial degeneration of the aorta 6. A number of studies have reported that apoptosis, autophagic cell death and pyroptosis are all involved in the loss of SMCs during AD 13, 50, 51. Ferroptosis has been reported to be involved in hyperlipidemia-associated vascular calcification 52, atherosclerosis 53, and the effects of cigarette smoke extract on SMCs 54, but whether ferroptosis participates in AD remains unknown. In the aortas of AD patients, we found that SLC7A11 and FSP1, key regulators of ferroptosis, were downregulated, which indicates that ferroptosis may contribute to SMC loss and AD development. Liproxstatin-1, a specific inhibitor of ferroptosis 55, was used to evaluate the role of ferroptosis in the pathological processes of AD. Surprisingly, liproxstatin-1 significantly inhibited BAPN-induced elastic fiber breakage, SMC loss, AD and rupture in mice. Recently, a study demonstrated that ferroptosis may be involved in the occurrence of AD by using bioinformatics analysis^56^, while our present study confirmed that ferroptosis of SMCs is an important cause of medial degeneration of the aorta and the progression of AD by conducting experiments in HASMCs, mice and specimens of patients with AD.

In conclusion, we found that the expression of METTL3 was obviously increased in the aortas of TAAD patients and was negatively correlated with the expression of the ferroptosis-regulating proteins SLC7A11 and FSP1. Furthermore, METTL3 promoted ferroptosis of SMCs by suppressing the expression of SLC7A11 and FSP1, and inhibiting ferroptosis largely reversed BAPN-induced AD in mice. Therefore, we have clarified that ferroptosis is a key mechanism involved in the development of AD and elucidated the RNA m^6^A mechanism that regulates ferroptosis in SMCs. Thus, targeting ferroptosis is expected to become a new strategy for delaying or treating AD.

## Supplementary Material

Supplementary figures and table.

## Abbreviations

SMCs: Smooth muscle cells
HASMCs: Human aortic SMCs
AD: Aortic dissection
TAAD: Stanford type A AD
SLC7A11: Solute carrier family 7 member 11
FSP1: Ferroptosis suppressor protein 1
GPX4: Glutathione peroxidase 4
BAPN: β-aminopropionitrile
METTL3: Methyltransferase-like 3
NADPH: Nicotinamide adenine dinucleotide phosphate
CoQ_10_: Coenzyme Q_10_
LC3: Microtubule-associated protein 1 light chain 3
m^6^A: N^6^-methyladenosine
H/R: Hypoxia/reoxygenation
ATG5: Autophagy related 5
NSCLC: Non-small cell lung cancer
AAA: Abdominal aortic aneurysm
AAD: Aortic aneurysm and dissection
IKE: Imidazole ketone erastin
shRNA: Short hairpin RNA
FBS: Fetal bovine serum
ACTD: Actinomycin D
CCK-8: Cell Counting Kit-8
LDH: Lactate dehydrogenase
OD: Optical density
RIPA: Radioimmunoprecipitation assay
PVDF: Polyvinylidene fluoride
HE: Hematoxylin and eosin
EVG: Elastin Verhoeff-van Gieson
EDTA: Ethylenediaminetetraacetic acid
PBS: Phosphate-buffered saline
MDA: Malondialdehyde
TBA: Thiobarbituric acid
ROS: Reactive oxygen species
PCA: Principal component analysis
DEGs: Differentially expressed genes
KEGG: Kyoto Encyclopedia of Genes and Genomes
SD: Standard deviation
HMOX1: Heme oxygenase 1
TFR: Transferrin receptor
4-HNE: 4-hydroxynonenal
YTHDC2: YT521-B homology domain-containing 2
ALKBH5: AlkB homolog 5
FTO: Fat mass and obesity-associated protein
WTAP: Wilms' tumor 1-associated protein
Klf5: Krüppel-like factor 5
WT: Wild-type
TFEB: Transcription factor EB
xCT: Cystine/glutamate antiporter
AIFM2: Apoptosis-inducing factor mitochondria-associated 2
