# Supplementary material for: Targeting Ferroptosis as a Novel Approach to Alleviate Aortic Dissection
Source: Int J Biol Sci. 2022 Jun 21;18(10):4118–34. doi: 10.7150/ijbs.72528 (PMC9274489; doi:10.7150/ijbs.72528)
Supplement: Supplementary file 1 — Supplementary figures and table. [file ijbsv18p4118s1.pdf]

Supplemental Table and Figures

Table S1. The clinical information of patients with TAAD and Non-AD.

Clinical Indicators	TAAD (n= 80)	Non-AD (n= 22)	*p* Value
**age (years), mean ± SD**	50.8±9.1	43.9±12.2	0.005
**Sex (male)**	67 (83.7)	19 (86.4)	0.765
**BMI (kg/m^2), mean ± SD**	25.8±3.9	22.3±3.9	<0.001
**Smoking (n, %)**	28 (35.0)	7 (31.8)	0.781
**Diabetes (n, %)**	3 (3.8)	5 (22.7)	0.003
**Hypertension (n, %)**	49 (61.2)	6 (27.3)	0.005
**Diameters of Aorta (mm)**			
**Ascending aorta**	47.7±7.6		
**Aortic arch**	36.5±5.0		
**Descending aorta**	31.8±5.0		
**CAD, median (range; IQR)**			
**cTnI (pg/mL)**	1259 (7.6-430.2)	9564 (19.1-1634)	0.003
**Myo (ng/mL)**	332.0 (49.8-391.8)	140.4 (30.2-203.6)	0.125
**CK-MB (ng/mL)**	8.5 (1.1-6.2)	39.5 (0.9-33.2)	0.026
**LVEF (%), mean ± SD**	59.3±5.0	27.6±12.9	<0.001
**D-Dimer (μg/mL), median (range; IQR)**	28.8 (5.5-29.8)	2.6 (0.7-2.5)	0.006

AD: Aortic dissection; TAAD: Stanford type A AD; SD: Standard deviation; BMI: Body mass index; CAD: Coronary artery disease; IQR: interquartile range; cTnI: Cardiac troponin I; Myo: Myoglobin; CK-MB: Creatine kinase-MB; LVEF: Left ventricular ejection fraction. The information of donors cannot be obtained due to confidentiality principles.

Supplemental Figure S1. Bioinformatics analysis demonstrated that ferroptosis is involved in the occurrence of AD in human.

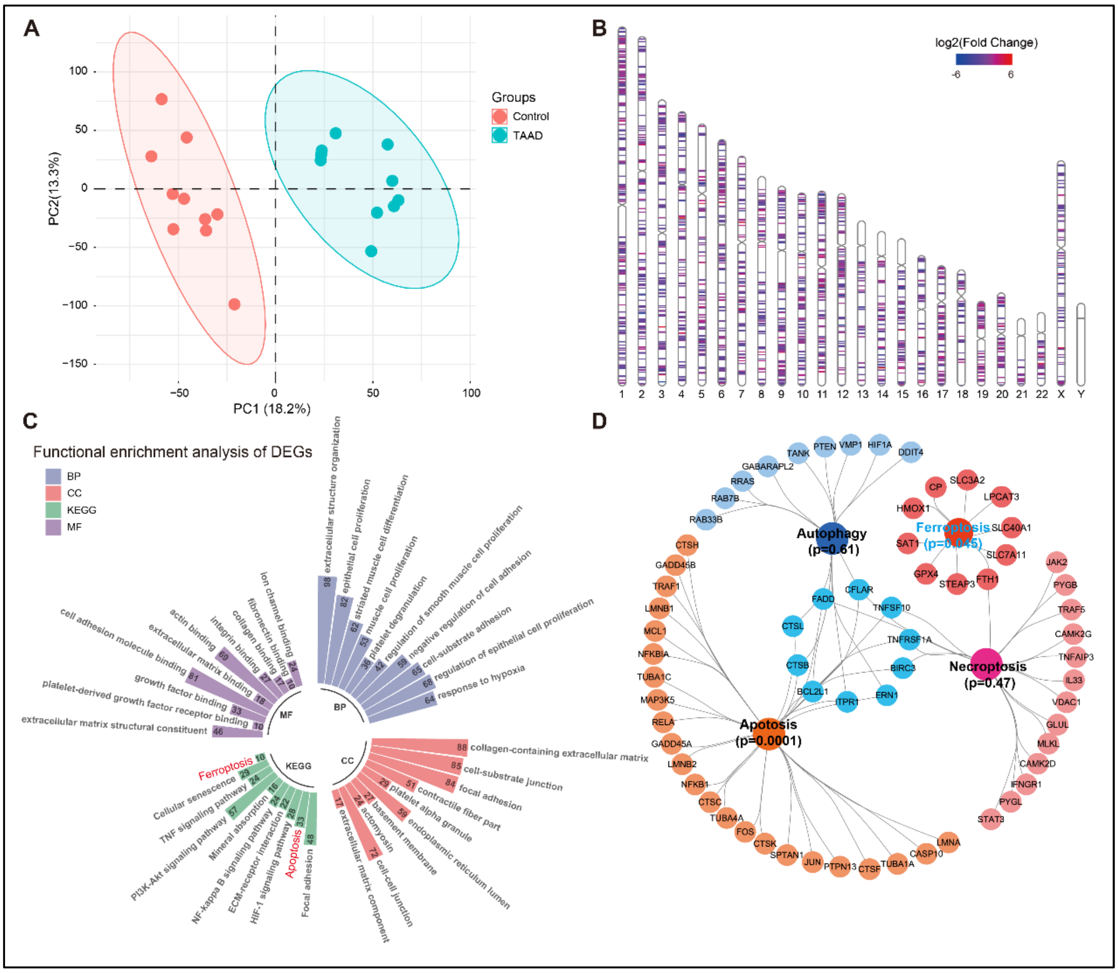

Figure S1. (A). Principal component analysis (PCA) of RNA-sequence dataset GSE153434 in aortas with or without AD in human. **(B).** Differentially expressed genes (|FC| ≥1.5 and p adjust value ≤ 0.05) of GSE153434 was shown in chromosomes. **(C).** Functional enrichment analysis of differentially expressed genes was performed based on the Gene Ontology (GO) database and Kyoto Encyclopedia of Genes and Genomes (KEGG) pathway. **(D).** The potential programmed cell death pathways and related differentially expressed genes during AD development based on GSE153434 dataset.

Supplemental Figure S2. The protein levels of SLC7A11, FSP1 and GPX4 were downregulated in the aortas of TAAD patients.

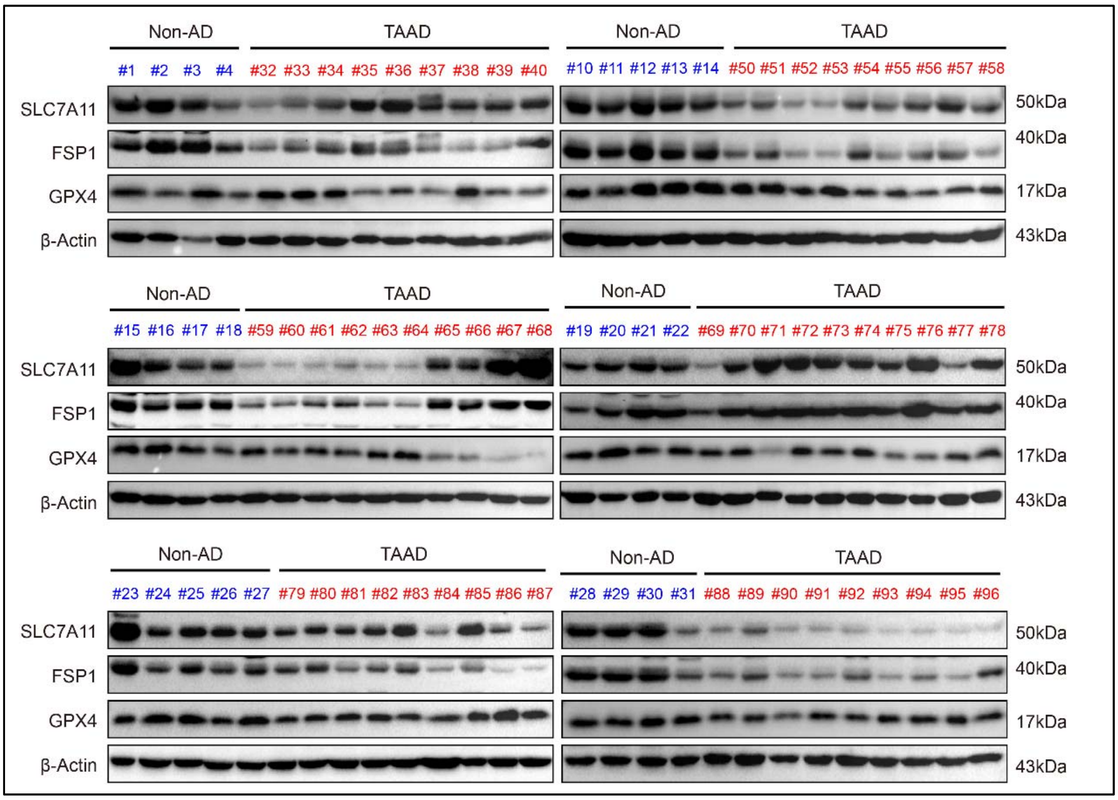

Figure S2. The blots of SLC7A11, FSP1, and GPX4 in aortas of non-AD and TAAD patients. β-actin served as the loading control.

Supplemental Figure S3. Protein levels of m^{6}A modulators in the aortas of non-AD and TAAD patients.

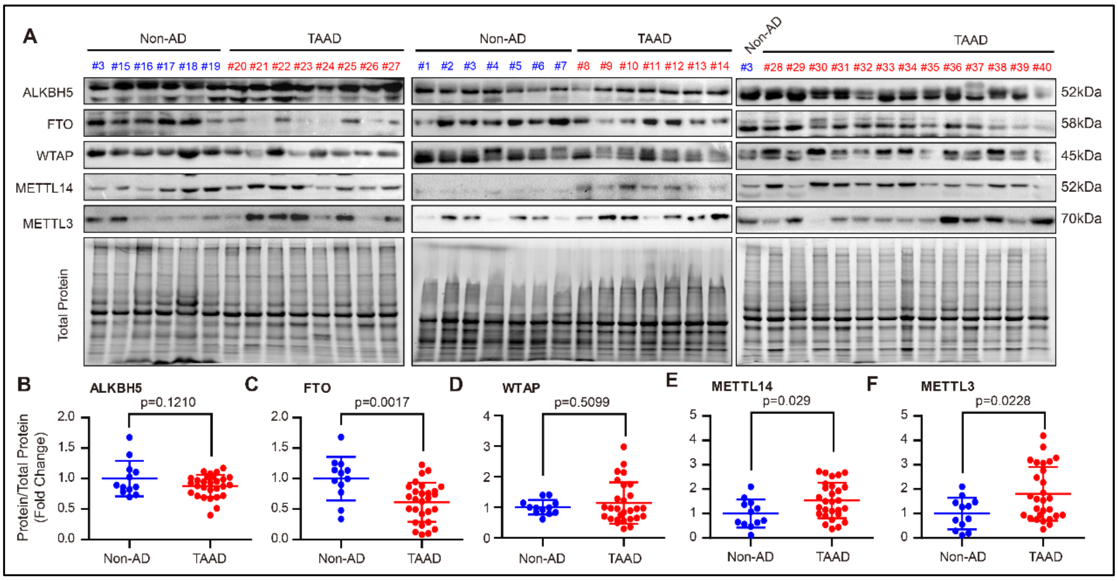

Figure S3. The protein levels of the m^{6}A demethylases ALKBH5 and FTO and the m^{6}A methyltransferase complex components WTAP, METTL14 and METTL3 were detected by western blot analysis in aortic tissues of non-AD and TAAD patients (n=12 non-AD and n=28 TAAD). **(A).** Western blots; **(B-F)**. Quantitative results of ALKBH5 (B), FTO (C), WTAP (D), METTL14 (E) and GPX4 (F) expression in the aorta. Total protein served as the loading control.

Supplemental Figure S4. Protein levels of METTL3 in the aortas of non-AD and TAAD patients.

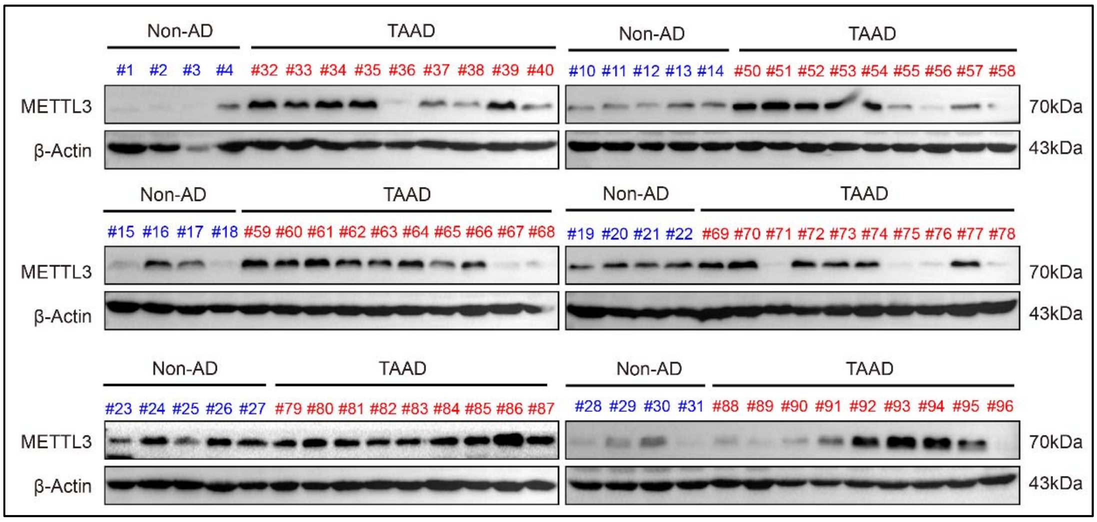

Figure S4. The blots of METTL3 in aortas of non-AD and TAAD patients. β-actin served as the loading control.

Supplemental Figure S5. The m^{6}A levels of SLC7A11 and FSP1 mRNA in human and mouse cell with METTL3 knockout or not in GEO datasets.

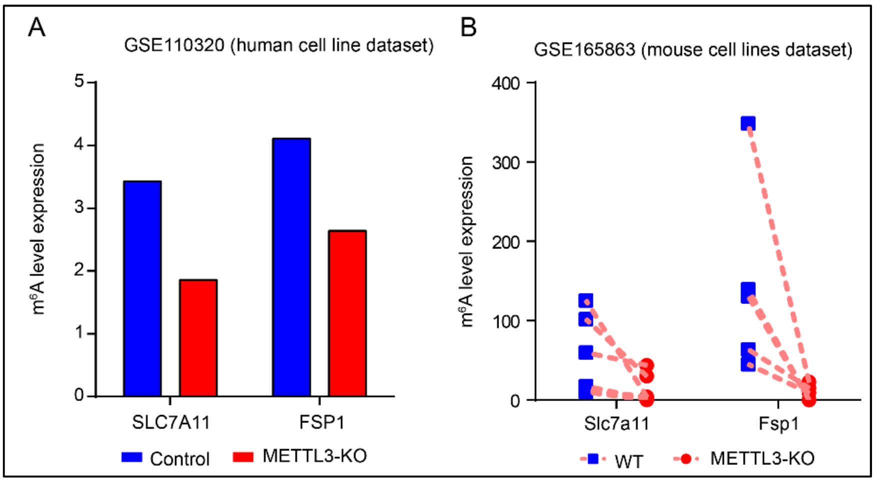

Figure S5. (A). The m^{6}A levels of SLC7A11 and FSP1 mRNA in human cell line with METTL3 knockout or not (GSE110320). **(B).** The m^{6}A levels of Slc7a11 and Fsp1 mRNA in mouse cell lines with METTL3 knockout or not (GSE165863).

Supplemental Figure S6. The protein levels of METTL3, SLC7A11 and FSP1.

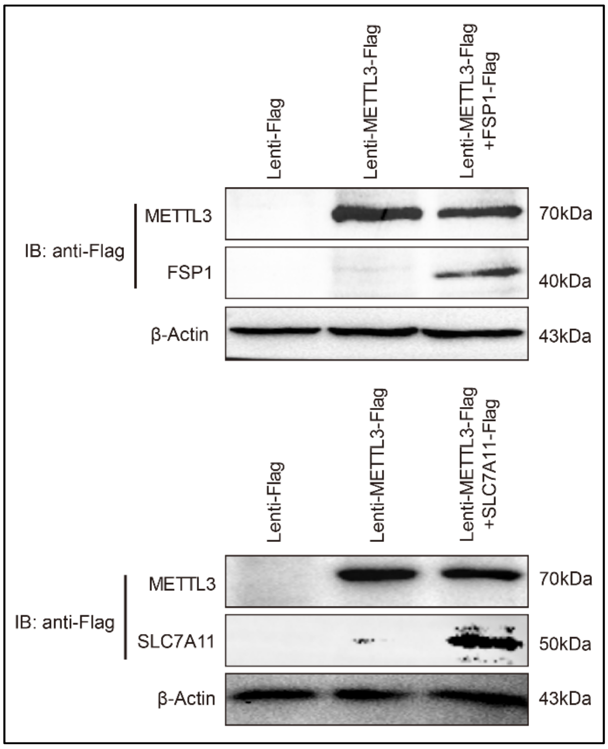

Figure S6. Representative western blots of METTL3, SLC7A11 and FSP1 in VSMCs with indicated lentivirus infection. β-actin served as the loading control.
